# Impact of integrating a postpartum family planning program into a community-based maternal and newborn health program on birth spacing and preterm birth in rural Bangladesh

**DOI:** 10.7189/jogh.08.020406

**Published:** 2018-12

**Authors:** Abdullah H Baqui, Salahuddin Ahmed, Nazma Begum, Rasheda Khanam, Diwakar Mohan, Meagan Harrison, Ahmed al Kabir, Catharine McKaig, Neal Brandes, Maureen Norton, Saifuddin Ahmed, for the Projahnmo Study Group in Bangladesh

**Affiliations:** 1 International Center for Maternal and Newborn Health, Department of International Health, Johns Hopkins Bloomberg School of Public Health, Baltimore, Maryland, USA; 2 Johns Hopkins University-Bangladesh, Dhaka, Bangladesh; 3 Research, Training and Management (RTM) International, Dhaka, Bangladesh; 4 Jhpiego, Baltimore, Maryland, USA; 5 US Agency for International Development, Washington, D.C., USA; 6 Department of Population, Family and reproductive Health, Johns Hopkins Bloomberg School of Public Health, Baltimore, Maryland, USA

## Abstract

**Background:**

Short birth intervals are associated with an increased risk of adverse perinatal outcomes. However, reduction of rates of short birth intervals is challenging in low-resource settings where majority of the women deliver at home with limited access to family planning services immediately after delivery. This study examines the feasibility of integrating a post-partum family planning intervention package within a community-based maternal and newborn health intervention package, and evaluates the impact of integration on reduction of rates of short birth intervals and preterm births.

**Methods:**

In a quasi-experimental trial design, unions with an average population of about 25 000 and a first level health facility were allocated to an intervention arm (n = 4) to receive integrated post-partum family planning and maternal and newborn health (PPFP-MNH) interventions, or to a control arm (n = 4) to receive the MNH interventions only. Trained community health workers were the primary outreach service providers in both study arms. The primary outcomes of interest were birth spacing and preterm births. We also examined if there were any unintended consequences of integration.

**Results:**

At baseline, short birth intervals of less than 24 months and preterm birth rates were similar among women in the intervention and control arms. Integrating PPFP into the MNH intervention package did not negatively influence maternal and neonatal outcomes; during the intervention period, there was no difference in community health workers’ home visit coverage or neonatal care practices between the two study arms. Compared to the control arm, women in the intervention arm had a 19% lower risk of short birth interval (adjusted relative risk (RR) = 0.81, 95% confidence interval (CI) = 0.69-0.95) and 21% lower risk of preterm birth (adjusted RR = 0.79; 95% CI = 0.63-0.99).

**Conclusions:**

Study findings demonstrate the feasibility and effectiveness of integrating PPFP interventions into a community based MNH intervention package. Thus, MNH programs should consider systematically integrating PPFP as a service component to improve pregnancy spacing and reduce the risk of preterm birth.

Short birth intervals are associated with increased risk of adverse maternal, perinatal, infant, and child health outcomes [1-4], ranging from stillbirth [5], small-for-gestational-age, low birth-weight to neonatal and maternal morbidity and mortality [6-8]. Three meta-analyses have found significant associations of short birth or inter-pregnancy intervals with preterm birth [1,9,10]. After reviewing evidence, experts have recommended to the World Health Organization (WHO) that couples wait 24 months after a live birth before attempting a pregnancy to reduce the risk of adverse maternal and newborn outcomes [11].

A recent analysis of Demographic and Health Survey (DHS) data from 21 low- and middle-income countries found that, in nine of those countries, 50 percent or more of non-first births occurred at intervals considered too short and in another nine countries, about 40 percent of non-first births occurred at intervals considered too short [12]. At the same time, studies suggest that, after childbirth, about 95 percent of the women in developing countries want to postpone pregnancy for at least two years, yet almost two-thirds of them do not use a contraceptive method [13]. Recent studies have found that, in low- and middle-income countries, postpartum women’s unmet need for contraception has not changed measurably over the past decade [12].

Promoting contraceptive use immediately after birth is considered an important family planning programmatic strategy for meeting postpartum women’s unmet need for contraception, preventing unintended pregnancies and short birth intervals. Postpartum women often resume sexual activity between 3-6 months after delivery, or sooner [14]. Yet, despite their preferences to delay the next pregnancy for at least two years, many women experience unintended, short interval pregnancies. Few women (or men) have knowledge of fertility and ovulation, and many lack understanding of the timing of fertility return after childbirth [15,16]. For non-lactating women, ovulation may occur as early as 45 days after childbirth, and in some cases, may occur before menses return [17]. Being not aware that fertility can return before menses, many women conceive again shortly after delivery [13].

Although, family planning is considered one of the four pillars of a safe motherhood program [18], contraceptive counseling and service deliveries are often not closely integrated with antenatal, delivery, and postpartum care services. Integration of family planning with maternal and child health programs has emerged as an attractive option in recent years as part of the “continuum of care” framework [19]. The periods of pregnancy, delivery, and postpartum are considered opportune for counseling women on the adoption of modern family planning methods due to frequent encounters with the health system [3,20,21]. Integration is anticipated to provide multiple opportunities to streamline service delivery and improve care at favorable and critical times for maximizing women’s reproductive health and the health of their children.

Although significant interest in integrating family planning with other health services emerged during the last 30 years, both for programmatic and political reasons [22], limited empirical evidence is available on the effectiveness of programs that integrate family planning with maternal, perinatal, and child health. Systematic reviews identified that most trials on the effect of integration of PPFP were conducted in developed countries [23-25]. There is a paucity of evidence from developing countries in terms of what intervention programs work best for PPFP in settings where most women deliver at home. Of the relatively very few studies on integration that have been conducted, most were limited by methodological quality including cross-sectional design, hospital based survey, non-family planning outcomes as main interest, short duration of observation, or lack the details of intervention for replication [24]. Overall, it is recognized that the evidence of the integration of postpartum family planning with other health services remains weak, and well-designed evaluation research is urgently needed [26].

This study presents the results of an integrated PPFP intervention package with an existing community-based MNH intervention package in a rural area of Bangladesh. Between 2007 and 2013, a quasi-experimental study, the *Healthy Fertility Study* (HFS), was undertaken by a research partnership known as Projahnmo Study Group in Bangladesh. Earlier, we presented the results of HFS on contraceptive prevalence rates (CPR), adoption patterns, and continuation rates during first 24 months postpartum by the study arms [27]. In this paper, we present the impact of the intervention in reducing rates of short birth intervals and preterm births.

## METHODS

### Study setting and design

**Figure 1:**
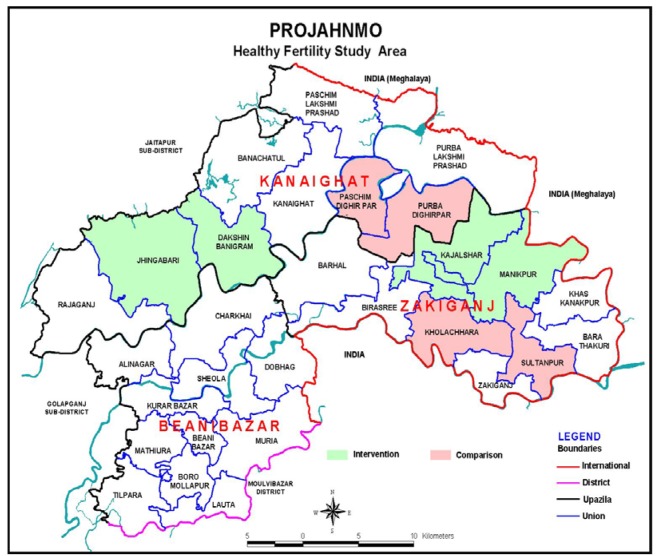
Map of the study area.

**Figure 2:**
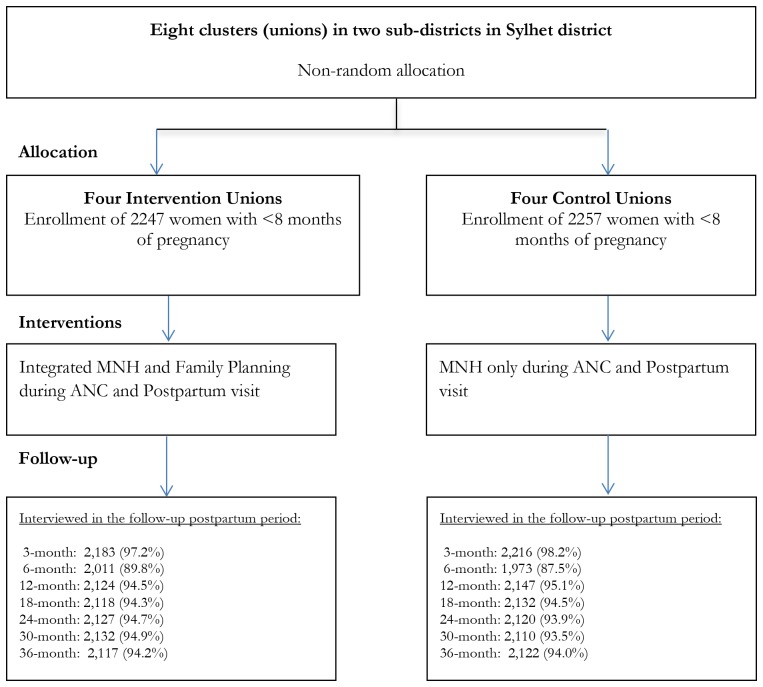
Trial profile.

This quasi-experimental community-based trial was conducted in eight *unions* (*unions* are the lowest administrative units in Bangladesh with an average population of about 25 000 and a health center known as Health and Family Welfare Center – H&FWC), in two sub-districts (Zakiganj and Kanaighat) of Sylhet District, Bangladesh. Four unions were allocated to the intervention arm (Manikpur, Kajalshar, Jhingabari and Dakshin Banigram) and the remaining four were allocated to the control arm (Sultanpur, Kholachara, Purbo Dighirpar and Paschim Dighirpar) (**Figure 1**). Between December 2007 and July 2009, the study enrolled 4504 pregnant women (2247 in the intervention arm and 2257 in the control arm) identified through two monthly home visits by community health workers (CHWs) (**Figure 2**). The sample size was calculated based on the baseline birth spacing rate in the study area. An earlier Projahnmo study documented that 16% of postpartum women in the study area had another birth outcome within 24 months of the index birth. We hypothesized that the proportion of women with a second birth outcome within 24 months will be 12% in the intervention arm, a decrease of 25% compared to the control arm. To measure a 25% decrease in the proportion of women with a short birth interval with 90% power and a 5% significance level would require a sample size of 1181 pregnant women per study arm. Taking into account an assumed design effect of 1.5 would increase the sample size to 1772. Assuming a 20% loss to follow-up would further increase the sample size to 2215 per study arm.

### Study site

Located in northeastern Bangladesh, Sylhet division is home of about 10 million of Bangladesh’s total population of 145 million [28]. Sylhet division falls behind all of the eight administrative divisions in Bangladesh for key MNH indicators [29,30]. Compared to the national contraceptive prevalence rate (CPR) of 61.2% and total fertility rate (TFR) of 2.3 per women, Sylhet experiences a CPR of 45% and a TFR of 3.1. The median birth interval is 37.6 months in Sylhet compared to 47.4 months nationally. [29] Within Sylhet, study unions were purposively selected.

### Intervention package

**Figure 3:**
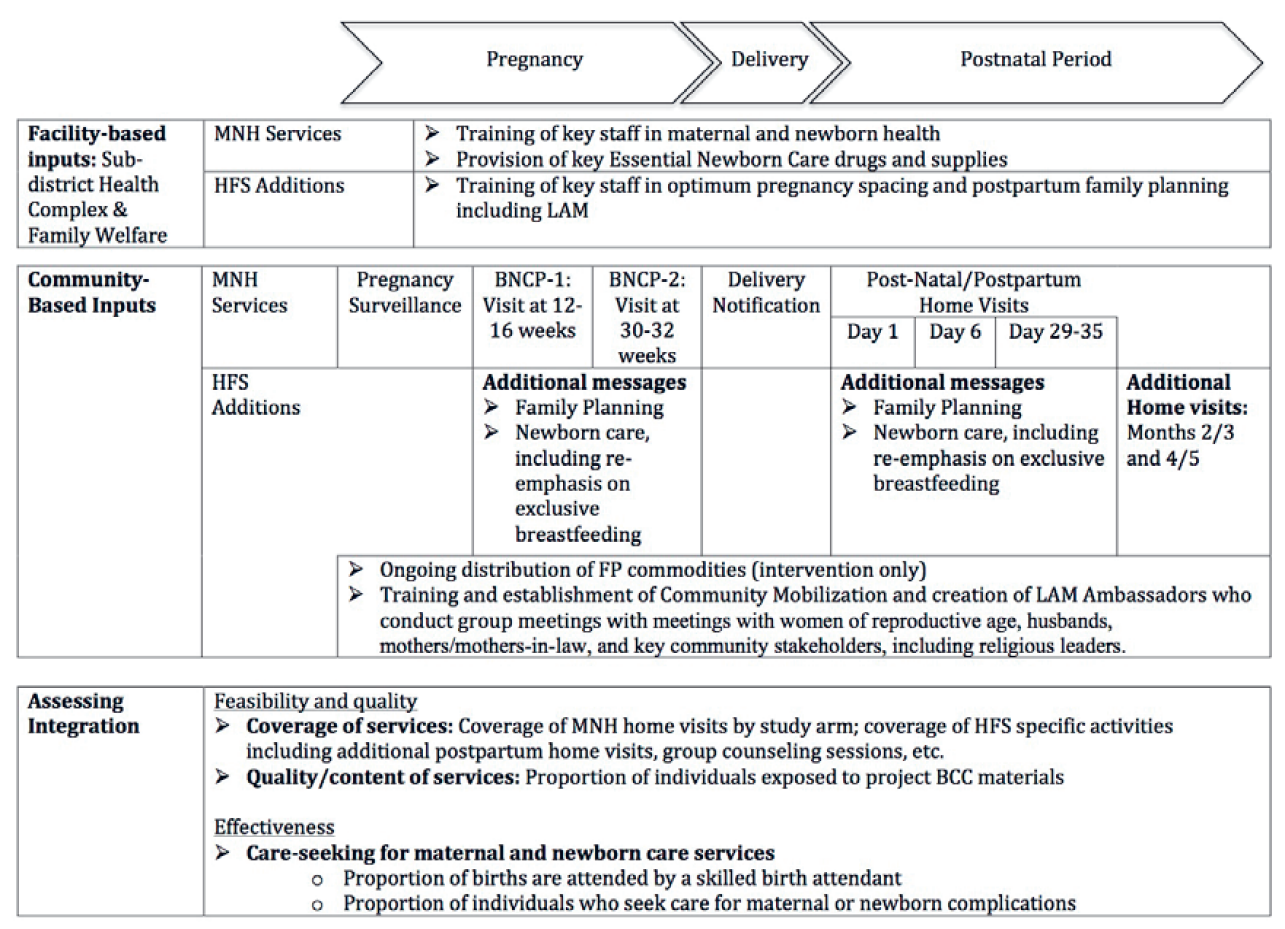
Integrated intervention in the Healthy Fertility Study.

The intervention packages of the Healthy Fertility Study has been described in detail previously [30]. **Figure 3** summarizes facility and community based MNH and PPFP interventions by study arm. A brief description of community and facility-based interventions by study arm is provided below.

### Community-based services

In both study arms, CHWs, each serving a population of about 4000 (about four villages), provided a platform of MNH services. This included 2-monthly home visits to identify pregnancies; two antenatal home visits and three postnatal home visits on first, third, and seventh days of childbirth; and identification and referral of sick neonates. In the intervention area, PPFP activities sought to build upon existing home visits to include (a) the integration of behavior change communication (BCC) messages on FP into planned antenatal and postpartum home visits (at 30-32 weeks of pregnancy; 6 days postpartum and 29-35 days postpartum) [27]; (b) on-going distribution of short term contraceptive methods, including pills and condoms and referrals for clinical methods such as IUDs; and (c) additional home visits at 2 or 3 and 4 or 5 months postpartum for a total of 5 postpartum visits. The visits at 4-5 months postpartum were intended to ensure that women were satisfied with their contraceptive method and to assist Lactational Amenorrhea Method (LAM) users to transition from LAM to another modern family planning method, since LAM is no longer effective after six months. PPFP activities also included training and establishment of Community Mobilizers (CMs) and voluntary “LAM Ambassadors” who collaboratively conducted group meetings with women of reproductive age, husbands, mothers and mothers-in-laws, and key community stakeholders including religious leaders.

Behavior change communication (BCC) messages emphasized the importance of birth spacing, for the health of the mother and child, including waiting at least 24 months after a live birth before conceiving again; the timing of postpartum return to fertility, including information on the fact that fertility can return before menses, and that women should not use return of menstruation as a signal to begin using contraception; use of the LAM, including timely transition at 5-6 months postpartum to another modern method; and the role of FP in improving maternal and newborn health [11]. One of the BCC materials was a leaflet that included a story and a pictorial on one side, and critical messages about return to fecundity on the other side. The leaflet and story were incorporated within home visits and community mobilization sessions as discussion aids [16].

### Facility-based inputs

Within the study area, efforts to strengthen H&FWCs and sub-district hospitals, which provide basic outpatient preventive and curative MNH services as well as family planning services, were also a vital part of study activities. In both study arms, government health facilities received essential newborn care drugs and supplies, and staff were trained in MNH. In the intervention area, study activities included provision of contraceptives and training of key staff in healthy pregnancy spacing and postpartum family planning including LAM.

### Data collection

A team of interviewers independent of the intervention collected data from the study women during the enrolment pregnancy (baseline), hereafter referred to as ‘index’ pregnancy and during the postpartum period through 36 months post-partum. Seven additional data collection visits were made at months 3, 6, 12, 18, 24, 30 and 36 after enrollment. The interviewers collected the following data: pregnancy, delivery and newborn care practices and survival status of the index child; program exposure (visits by CHWs and CMs), attendance at community meetings, contraceptive use history, subsequent pregnancy incidences and outcomes during the follow-up period. The survey rounds at three and six months also collected data on resumption of menstrual period, sexual activity resumption, and breastfeeding. In the case of stillbirths or neonatal deaths, women were interviewed with a shorter form without any reference to postpartum contraceptive use; these women were excluded from the analysis. The current study results are based on data analyses from all survey rounds. The trial profile shows the number of women interviewed and coverage rate of surveys in each round (**Figure 2**).

### Statistical analyses

To assess the differences between the study arms at baseline, we calculated means and proportions of selected background characteristics and compared them with the Rao-Scott second order corrected χ^2^ tests for categorical variables and adjusted Wald-statistics for continuous variables; these statistical methods were used to account for variances in clustered data [31,32]. A wealth index score was constructed for each household based on household durable goods and type of household (ie, materials used to construct wall, roof, and floor of the house) using principal component analysis. Households were ranked according to the total wealth score and then divided into wealth quintiles. An intention to treat analysis was conducted in which all observations were included irrespective of exposure to intervention.

We estimated the differences in the risk of short birth intervals <24 months and preterm births between the study arms using log-binomial regression models, adjusting for confounding covariates. The following confounding covariates, identified from bivariate analysis and the literature, were included in regression models: age, parity, socioeconomic status, woman’s education, husband’s education and religion. Additionally, fertility desire and previous contraceptive use before the index pregnancy were included in the birth interval analysis. Since women who had experienced a shorter birth interval or preterm birth earlier might experience them again, we adjusted for baseline differences in birth intervals and preterm birth rate in our analyses of short birth interval and preterm birth during the intervention period.

We also examined the differences in the distribution of birth intervals (birth-to-birth) between the intervention and control arms by Kaplan-Meier life-table method and hazards regression model. Since our preliminary analysis suggested that the data violated the proportionality assumption of the Cox model, we used the parametric hazards models with Weibull distribution. Robust standard errors were used in all regression analyses to account for the cluster nature of the data.

The study was approved by the Johns Hopkins Bloomberg School of Public Health Institutional Review Board and the Bangladesh Medical Research Council Ethics Committee. Study participants included married women of reproductive age between 15-49 years, who provided informed verbal consent for the study participation. The study was limited to pregnant women, and those pregnant women aged below 18 years were considered “emancipated minors” because they were legally married and experienced pregnancy, or had given birth and were enduring adult responsibilities. As such, both the Johns Hopkins Bloomberg School of Public Health IRB and the Bangladesh Medical Research Council IRB approved the method of taking of consent of the 15-17 year-old pregnant women without parent/guardian consent. The consent process was documented in a printed consent form. All consent forms were signed and dated by the consent taker/ interviewer.

### Role of the funding source

The study sponsors had no role in the study design, data collection, analysis, interpretation, or dissemination, or in the decision to submit this paper for publication. The corresponding author has full access to all the data in the study and had the final responsibility for the decision to submit for publication.

## RESULTS

The study enrolled 2247 and 2257 pregnant women in the intervention and control arms respectively (**Figure 2**). The baseline sample characteristics were similar in terms of women’s age, husbands’ education, parity and religion (**Table 1**). However, women in the intervention arm had higher mean years of education (4.5 vs 4.1 years) and better household economic status compared to women in the control arm.

**Table 1:** Selected baseline characteristics of women at enrollment by study arms

	Intervention		Control			*P*-value*		
	**N**	**%/mean**	**N**		**%/mean**			
**Women’s age (years):**								
15-19	204	9.1%		147	6.5%			
20-24	644	28.7%		673	29.8%			
25-29	757	33.7%		760	33.7%			
30-34	395	17.6%		490	21.7%			
35+	247	11.0%		187	8.3%			
Mean (95% CI)	2247	26.5 (26.1-26.9)		2257	26.6 (26.3-26.9)		0.764	
**Women’s education:**								
No schooling	729	32.4%		811	35.9%			
Primary (1-5 years)	692	30.8%		749	33.2%			
Secondary and above (>5 years)	826	36.8%		697	30.9%			
Mean (95% CI)	2247	4.5 (4.2-4.8)		2257	4.1 (3.9-4.3)		0.025	
**Husbands’ education:**								
No schooling	961	42.8%		873	38.7%			
Primary (1-5 years)	621	27.6%		766	33.9%			
Secondary and above (>5 years)	665	29.6%		618	27.4%			
Mean (95% CI)	2247	4.1(4.2-4.8)		2257	4.0 (3.7-4.3)		0.768	
**Parity:**								
Primigravida	573	25.5%		567	25.1%			
1-2	864	38.5%		862	38.2%			
3-4	516	23.0%		502	22.2%			
5+	294	13.1%		326	14.4%			
Mean (95% CI)	2247	2.2 (2.1-2.3)		2257	2.2 (2.1-2.3)		0.655	
**Religion:**								
Muslim	2135	95.0		2080	92.2		0.243	
Hindu/Other	112	5.0		177	7.8			
**Wealth quintile:**								
Lowest	407	18.1%		495	21.9%		<0.001	
Second	380	16.9%		518	23.0%			
Middle	440	19.6%		461	20.4%			
Fourth	510	22.7%		391	17.3%			
Highest	510	22.7%		392	17.4%			

CI – confidence interval

*P-values are adjusted for clustering effect at community level (design-effect >1) with Rao-Scott second order corrected χ^2^-tests for categorical variables and with Taylor linearization method for continuous variables.

A concern related to FP-MNCH integration activities is that while adding family planning may improve FP outcomes, the addition of new tasks and activities may undermine MNH service delivery performance and affect outcomes. Our analyses indicate that adding family planning to the maternal and neonatal health intervention package did not negatively influence MNH coverage or selected newborn care practices. **Table 2** shows data on CHW’s antenatal and postnatal home visit coverage and selected newborn care practices as proxy indicators for feasibility of integration and of compliance with MNH advice provided by CHWs during home visit counseling and community mobilization meetings. The antenatal visit coverage was almost universal in both intervention and control arms (99.4% and 99.6%, respectively). Postpartum visits by CHWs, as reported by the mothers at the first follow-up survey round at 3^rd^ month following the birth of the index child, were slightly higher in the intervention arm (95.6% vs 93.0%).

**Table 2:** Community Health Workers home visit coverage and newborn care practices for the index birth

	Intervention arm	Control arm	*P*-value
CHW visit coverage:*			
ANC visit	2163/2183 (99.4)	2207/2216 (99.6)	0.322
PP visit	2087/2183 (95.6)	2061/2216 (93.0)	0.001
Timing of wrapping the baby after delivery (home delivery only):			
<10 min	963/1863 (51.7)	873/1894 (46.1)	<0.001
≥10 min	891/1863 (47.8)	1020/1894 (53.9)	
Don’t remember	9/1863 (0.5)	1/1894 (0.5)	
Initiation of breastfeeding:			
Within 30 min	1082/1863 (58.1)	937/1894 (49.5)	<0.001
After 30 min	753/1863 (40.4)	948/1894 (50.0)	
Don’t remember	28/1863 (1.5)	9/1894 (0.5)	

CHW – community health worker, ANC – antenatal care, PP – postpartum

*As reported in the 3^rd^ month follow-up visit.

Among those women who delivered at home (1863 and 1894 in the intervention and control arms, respectively), a significantly higher proportion in the intervention arm wrapped the baby within 10 minutes to prevent thermal loss (51.7%), compared to the women in control arm (46.1%). Similarly, the rate of early initiation of breastfeeding was about 9% higher in the intervention arm compared to the control arm (58.1% vs 49.5%, *P* < 0.001).

To examine the impact of integration of PPFP and MNH program on birth intervals and birth outcomes, we compared the rates of short birth intervals between the intervention and control arms for both the index children at enrollment and for subsequent births during the 36-month follow-up period. **Table 3** shows that the reported rates of short birth interval of less than 24 months among women who had a live birth were not different between the intervention and control arms (17.9% and 16.2%, respectively; *P* = 0.254) for the index pregnancies (at baseline). However, during the intervention period, among women who were observed for at least 24 months postpartum (2107 women in intervention and 2094 women control arms), a significantly lower proportion of women in the intervention arm (15.4%) had a shorter birth interval of less than 24 months compared to women in the control arm (18.6%). The multivariable log-binomial regression analysis, adjusted for baseline differences in birth interval among the index children and other demographic and socioeconomic covariates, shows that the risk of short birth interval was 19% lower in the intervention arm than the control arm (adjusted relative risk (RR) = 0.81; 95% CI = 0.69-0.95).

**Table 3:** Baseline differences in birth interval and preterm birth and effect of intervention on birth intervals and preterm births during the 36 months follow-up period

	At baseline (index children)				During 36-month follow-up period (subsequent children)			
	**Intervention**	**Control**	*P*-value*	**RR (95% CI)**	**Intervention**	**Control**	*P*-value*	**RR (95% CI)**
Birth Interval (N) ^§^	2168†	2156†			2107‡	2094‡		
Primigravida	46.2	45.6	-	-	-	-		-
<24 months	17.9	16.2	0.25	1.13 (0.99-1.29)	15.4	18.6	0.01	0.81 (0.7-0.95)
24+ months (or those who did not have a subsequent birth)	36.0	38.2		1.0	84.6	81.4		1.0
Preterm births¶	2168	2156			603	537		
Yes	22.9	25.5	0.06	0.93 (.83-1.04)	20.3	26.0	0.04	0.79 (.63-.99)
No	77.1	74.5			79.7	74.0		1.0

RR – relative risk, CI – confidence interval

*P-values are based on the Rao-Scott second-order corrected χ^2^ for the cross-tabulation analyses.

†Limited to women who had a live birth.

‡Limited to women who were followed-up for at least 24 months.

§Birth spacing analysis adjusted for age, parity, socioeconomic status, women’s education, husband’s education, religion, fertility desire and previous contraceptive use before the index pregnancy.

¶Preterm analysis of the subsequent children additionally adjusted for the preterm status of the index child.

**Figure 4:**
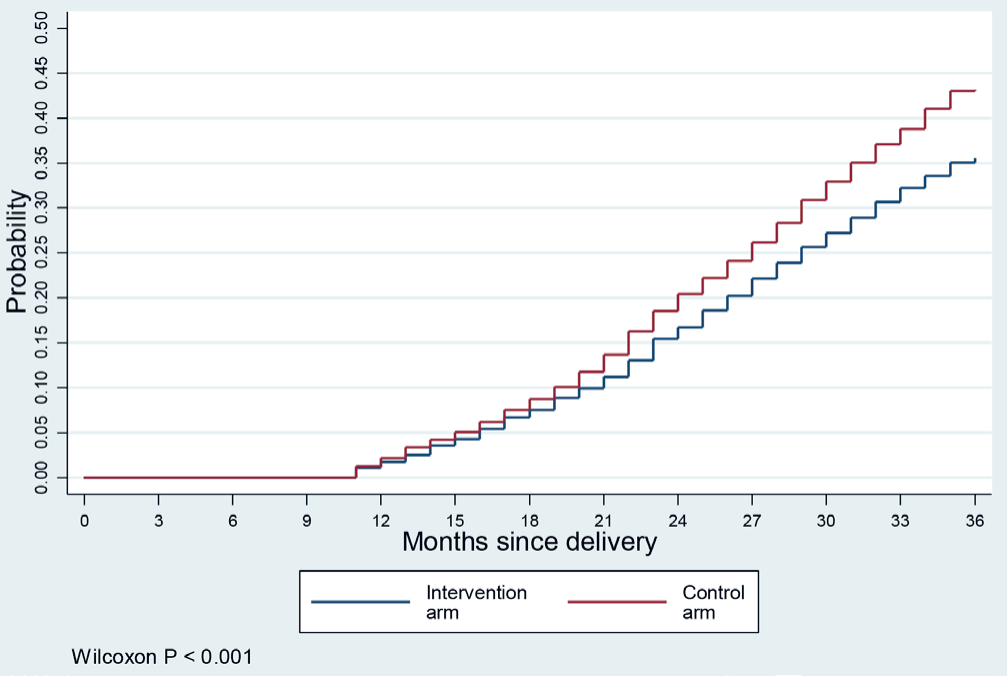
Birth-birth intervals.

**Figure 4** shows the cumulative probabilities of another birth in the 36 months follow-up period after the delivery of index child. The data graphed suggests that the risk of subsequent pregnancy increased steadily during the observed postpartum period at a constant rate. However, the risk of subsequent pregnancy was significantly lower in the intervention arm. In this analysis we also excluded women who were not observed for at least 24 months. As a sensitivity analysis, we also applied a hazards regression model with all samples (n = 4324, results are not shown) with Weibull distribution, which showed a hazard of short birth interval 24% lower in the intervention arm (hazards ratio (HR) = 0.76; 95% CI = 0.67-0.86).

At enrollment, the preterm birth rate among the index children, estimated based on mothers’ reported last menstrual period (LMP), was slightly lower in the intervention arm (22.9%) compared to the control arm (25.5%); however, this difference was not statistically significant (*P* > 0.05). The adjusted relative risk (RR) was also not statistically significant (RR = 0.93; 95% CI = 0.83-1.04). Among 1140 children who were born alive during the follow-up period, 20.3% were preterm births in the intervention arm, compared to 26.0% in the control arm (*P* < 0.05). Controlling for baseline differences in the distribution of preterm births among the index children and other demographic and socioeconomic variables, the adjusted risk of preterm births was 21% lower in the intervention arm (RR = 0.79; 95% CI = 0.63-0.99).

## DISCUSSION

This quasi-experimental study was conducted to evaluate the feasibility and potential impact of delivering a package of PPFP interventions including behavior change communications and selected services, integrating within a community-based MNH program in a rural population in Bangladesh. The CHW home visit coverage was similar in both arms suggesting that integration was feasible. Beyond coverage of basic services, to assess feasibility of integration we also considered whether there might have been unintended adverse consequences associated with integration on MNH indicators and there were none. We assessed the impact of the integrated package on contraceptive use prevalence, on birth spacing, and rates of preterm births. Earlier, we presented the results of the impact of the integrated PPFP-MNH program on contraceptive use behavior; the contraceptive prevalence rate was significantly higher in the intervention arm than in the control arm throughout the follow-up period [33]. We now demonstrate that the women in the intervention arm had significantly lower rates of short birth intervals and preterm births, compared to women in the control arm. The risks of shorter birth intervals and preterm birth were 19.0% and 21.0% lower, respectively, in the intervention women compared to the control women. The integrated package was associated with significantly increased cumulative probability of modern method adoption through the 36 months postpartum period, preventing pregnancies occurring in the time period associated with the highest risk of preterm birth in the next pregnancy [1,9,10].

A recent review prepared for the US Agency for International Development examined the evidence for MNCHN-FP integration and provided evidence for integration, discussed factors that promote or inhibit program effectiveness, discussed best practices and lessons learned, and identified recommendations for program planners, policy makers, and researchers [24]. A total of 36 peer-reviewed articles were included in this review, and they reported on 29 distinct interventions. Ten studies were conducted in Sub-Saharan Africa; nine in South Asia; three in Latin America; two in East Asia; and one each in Russia, Syria, Italy, US, and Australia. The review documented that integrating MNCH and FP services was feasible across a variety of integration models, settings, and target populations. Most studies reported that integration had a positive impact on reported outcomes; however many studies also reported mixed effects or no effect on some outcomes. No studies reported negative outcomes, which could be the result of publication bias, as studies are more likely to be published if they have positive results. Eleven of the 15 studies that measured use of MNCH and FP services reported an increased utilization of services due to integration. Measures of effectiveness included health and behavioral outcomes. The most commonly reported behavioral outcome was family planning use. Of 26 studies reporting this outcome, 19 found an increase in family planning use as a result of the integrated intervention, whereas seven found mixed or no effect. The most commonly reported health outcome was subsequent pregnancy. Of ten studies reporting this outcome, four found a decrease in pregnancy as a result of the integrated intervention, whereas six found mixed or no effect. Most studies used designs that were less than optimal such as before-after or serial cross-sectional.

In contrast, our study showed significantly increased uptake of family planning, lower risk of high-risk short birth intervals, and lower risk of preterm birth. We hypothesize that the program elements that were critical in achieving the outcomes include: 1) targeted services and messages to women during antenatal and postpartum home visits; 2) social and behavioral change communication messages conveyed as an integral element of service delivery - the messages, especially on the importance of waiting at least 24 months after a live birth before conceiving again, and that fecundity could return before menses, facilitated the adoption of healthier behaviors; and 3) counseling on and use of the LAM and transition to another modern method at 6 months - use of this highly culturally acceptable modern method [34] facilitated contraceptive use immediately after delivery and thus protected women from conceiving again during the time period of greatest risk for preterm birth if conception were to happen during this time.

Short birth intervals have been shown to be associated with increased risk of several adverse perinatal outcomes including preterm births [1]. However, there are disagreements on whether the relationship is causal or due to confounding by other risk factors such as socioeconomic status, other life style factors or other underlying disorders. Because we have adjusted for potential confounders as well as prior risk of preterm birth, which should be a reasonable proxy for underlying factors such as maternal nutrition, infection or genetic variations, we consider that the intervention is the likely explanation for the observed lower risk of preterm birth in the intervention area. The substantially lower risk of preterm birth is of significant public health importance since an estimated one million newborns die each year globally due to preterm birth related complications [35].

We believe that the intervention described in this paper is scalable. It reflects WHO recommendations for integrating family planning counseling, services and referrals into multiple MNCH service contact points in the health system. These include: antenatal care, labor and delivery, pre-discharge, postnatal care, well baby care, and immunization services [36]. This intervention does not necessarily depend on use of Community Health Workers or postnatal home visits, although this approach may be appropriate for rural, underserved areas.

We have implemented and assessed the impact of a community-based approach to implementing the WHO recommendations. In the intervention that we describe, family planning counseling, services, and referrals were integrated into three MNH points of contact: antenatal care, postnatal MNCH home visits at day 6, and postnatal visits at 29-35 days postpartum. We added two additional family planning visits at 2-3 and 4-5 months postpartum in our MNH intervention package. These visits were added to ensure that the woman was satisfied with her method and to assist LAM users to transition to another modern family planning method. Other variations of this model are possible.

For example, the WHO recommendations for family planning integration can be implemented in facilities. Pfitzer et al describe the scale-up of facility-based postpartum family planning services in six countries using a modified version of the intervention package [37]. In the six programs Pfitzer et al describe, family planning counseling and contraceptive services were integrated into three health system contact points: antenatal care, labor, and postpartum [38]. Immunization could provide an additional contact point. In some countries, progress is being made in linking postpartum family planning counseling and referrals with routine immunization [39,40]. After a review of the evidence, multiple international organizations now consider this a high impact practice in family planning [41].

There is a dearth of evidence in the published literature on postpartum family planning implementation and on models for effective counseling, including the timing and periodicity of counseling [38]. Health planners are endeavoring to scale-up quality maternal and neonatal care in low-resource settings [42]. The intervention described in our paper presents one approach to including FP counseling and services in the MNCH scale-up process, and the impact that might be achieved through integrated services.

Our study has several limitations. It was a quasi-experimental study as opposed to randomized controlled trial. However, our analysis is adjusted for all measured confounders. In addition, we conducted the short birth interval analysis adjusting for prior risk of short birth intervals and preterm birth analysis adjusting for prior risk of preterm birth. These adjustments should take into account the effects of unobserved heterogeneity between groups. The sample size of our study was not large enough and follow-up was limited to 36 months, which did not allow us to examine the effect of the intervention on child survival. The preterm birth rate was slightly but not significantly higher at baseline in the control arm; the analysis of intervention effect was, however, adjusted for prior risk of preterm birth. The preterm status was determined based on mothers’ reported last menstrual period (LMP), which may be subject to recall error leading to misclassifications. However, we conducted 2-montly home visits and prospectively collected LMP data from all women. Therefore, the recall period was short and any potential misclassification of term and preterm status should be minimal.

This was a community-based prospective study in a developing country setting. The study was conducted in a well-organized field site with a track record of conducting high quality and high impact studies [43,44]. This presumably minimized any potential measurement errors. We recommend that MNH programs should consider systematically integrating PPFP as it benefits from early and sustained FP use, reduced risk of short birth intervals, and fewer preterm births.
